# A retrospective chart review study to describe selected zoonotic and arboviral etiologies in hospitalized febrile patients in the Republic of Armenia

**DOI:** 10.1186/s12879-016-1764-z

**Published:** 2016-08-24

**Authors:** Lusine Paronyan, Eduard Zardaryan, Vahe Bakunts, Zaruhi Gevorgyan, Vigen Asoyan, Hripsime Apresyan, Alvard Hovhannisyan, Karo Palayan, Christian T. Bautista, Tinatin Kuchuloria, Robert G. Rivard

**Affiliations:** 1 National Center for Disease Control and Prevention, Ministry of Health of Armenia, Yerevan, Armenia; 2 Nork Infectious Diseases Clinical Hospital, Ministry of Health of Armenia, Yerevan, Armenia; 3 Walter Reed Army Institute of Research, Silver Spring, MD USA; 4 US Army Medical Research Institute of Infectious Diseases, Fort Detrick, MD USA

**Keywords:** Armenia, Febrile, Zoonotic, Hospital, Epidemiology

## Abstract

**Background:**

Scant information is available on the infectious causes of febrile illnesses in Armenia. The goal of this study was to describe the most common causes, with a focus on zoonotic and arboviral infections and related epidemiological and clinical patterns for hospitalized patients with febrile illnesses of infectious origin admitted to Nork Infectious Diseases Clinical Hospital, the referral center for infectious diseases in the capital city, Yerevan.

**Method:**

A chart review study was conducted in 2014. Data were abstracted from medical charts of adults (≥18 years) with a fever (≥38 °C), who were hospitalized (for ≥24 h) in 2010–2012.

**Results:**

Of the 600 patients whose charts were analyzed, 76 % were from Yerevan and 51 % were male; the mean age (± standard deviation) was 35.5 (±16) years. Livestock exposure was recorded in 5 % of charts. Consumption of undercooked meat and unpasteurized dairy products were reported in 11 and 8 % of charts, respectively. Intestinal infections (51 %) were the most frequently reported final medical diagnoses, followed by diseases of the respiratory system (11 %), infectious mononucleosis (9.5 %), chickenpox (8.3 %), brucellosis (8.3 %), viral hepatitis (3.2 %), and erysipelas (1.5 %). Reviewed medical charts included two cases of fever of unknown origin (FUO), two cutaneous anthrax cases, two leptospirosis cases, three imported malaria cases, one case of rickettsiosis, and one case of rabies. Engagement in agricultural activities, exposure to animals, consumption of raw or unpasteurized milk, and male gender were significantly associated with brucellosis.

**Conclusion:**

Our analysis indicated that brucellosis was the most frequently reported zoonotic disease among hospitalized febrile patients. Overall, these study results suggest that zoonotic and arboviral infections were not common etiologies among febrile adult patients admitted to the Nork Infectious Diseases Clinical Hospital in Armenia.

## Background

Armenia, located in the South Caucasus region, has an estimated population of 2.9 million [1]. Approximately 65 % of the population lives in urban areas, and the literacy rate among adults is almost 100 %. The country is divided into 11 regional government areas called marzes (ten regions plus Yerevan, the capital city) [2]. In Armenia, the National Center of Disease Control and Prevention (NCDCP), under the supervision of the Ministry of Health (MoH), is responsible for the control and prevention of communicable and noncommunicable diseases [3]. The NCDCP also develops and implements disease control policies and conducts epidemiological and environmental research to monitor and identify harmful factors that may affect the health of the population.

Available literature indicates that Q fever, plague, and sandfly fever were reported in Armenia in the late 1970s [4]. According to the NCDCP, the mortality rate from infectious and parasitic diseases in Armenia decreased from 12.4 deaths per 100,000 persons in 1990 to 9.2 in 2009 [5]. Unpublished MoH data indicates that acute respiratory infections and enteric infections were the main infectious diseases reported in Armenia during 2012–2013. During this period, 446, 30, and 2 cases of brucellosis, anthrax, and tularemia, respectively, were reported. The annual incidence rate of brucellosis was seven cases per 100,000 persons, with most cases occurring in the southern parts of Armenia and in rural areas adjacent to the neighboring country of Georgia. In 2006, a large entomological survey (64,567 mosquitoes and 45,180 *Ixodes* ticks), conducted by the Armenian Institute of Epidemiology, Virology, and Medical Parasitology, identified 125 distinct strains of arboviruses, including West Nile fever virus and tick-borne encephalitis virus [6]. In recent years, several studies have reported that endemic and emerging zoonotic infections, such as anthrax, leptospirosis, leishmaniasis, brucellosis, Crimean–Congo hemorrhagic fever, and hantavirus infection, are circulating in the South Caucasus region [7–13]. Despite the growing evidence suggesting that Armenian residents may be exposed to these zoonotic infections, little is known about the probable causes of febrile illness in the country. Therefore, a chart review study was undertaken to obtain information on the etiology, epidemiology, and clinical features among febrile patients admitted to the Nork Infectious Diseases Clinical Hospital. The Nork Hospital is the main referral center for pediatric and adult infectious diseases located in Yerevan, the capital city.

## Methods

The study population was composed of hospitalized febrile patients admitted to the Nork Hospital between January 2010 and December 2012. Medical charts of inpatients with the following characteristics were eligible and selected for data abstraction: 1) 18 years of age or older; 2) fever (axillary temperature ≥38 °C) at hospital admission; and 3) hospitalization for at least for 24 h.

For this study, a standardized questionnaire was developed based on the medical charts used at the Nork Hospital. To describe the study population, questions on demographic, epidemiological, and clinical characteristics were developed. Information on exposure history and factors that influence the risk of transmission of zoonotic and arboviral infections were also collected through the questionnaire. The questionnaire was initially developed in English, and then translated into Armenian. The Nork Hospital study team extracted data from the medical charts to complete the questionnaires, which were then sent to the NCDCP for data entry and analysis. For data entry quality control, 20 % of the questionnaires were double-entered, compared, and reconciled. In addition, we ensured that values for study variables were within the response range and conducted a database consistency check.

For sample size calculation, the Nork study team indicated that, of the 5000–6000 patients admitted to the hospital annually, approximately 250–300 patients met inclusion criteria. A convenience sampling of 600 medical charts over the study period (200 charts per year) were selected for data extraction, analysis, and reporting. In order to study seasonal variations or other changes over time, we extracted (where available) on average the first 20–35 charts per month that met the study criteria.

The following microbiological laboratory assays were used for diagnosis during the study period: Wright and Huddleston as well as Rose–Bengal serologic assays for brucellosis; commercial enzyme-linked immunosorbent assay (ELISA) for leptospirosis, hepatitis C, B and A viruses (HCV, HBV, HAV), human immunodeficiency virus (HIV), *B. anthracis*, herpes simplex virus (HSV), cytomegalovirus (CMV), and Epstein–Barr virus (EBV); agglutination assay was available for *Rickettsia* spp. Blood culture was conducted routinely for fever of unknown origin (FUO) cases and when bacteremia was suspected. In addition to the above-mentioned serologic assays, bacteriological and molecular polymerase chain reaction (PCR) assays were used to isolate *B. anthracis* culture and detect genetic material of the bacteria from the skin swab sample. Chickenpox and rabies was diagnosed based on the clinical manifestation combined with the supportive epidemiological information. Malaria was diagnosed based on the thick and thin blood smear microscopy. ELISA and bone marrow biopsy sample microscopy were used in case of suspected leishmaniasis. Bacteriological investigations of stool were conducted, and immunoenzyme assays and microscopy were used to diagnose bacterial, viral, and parasitic diseases of the gastrointestinal tract. Additionally, PCR was available for HSV-1 and −2, HBV, HCV, EBV, CMV, as well as for *Toxoplasma gondii*.

Statistical analyses were carried out using Epi Info version 3.5.6 and the Statistical Package for Social Science (SPSS) version 19. The International Classification of Diseases, Tenth Revision was used to code and classify medical diagnoses registered in patient charts. Maps were created using the GIS program ArcMap version 10.1, the geographic projected coordinate system, and WGS 1984 Universal Transverse Mercator Zone 38°N.

This study protocol was approved by institutional review boards at the U.S. Army Medical Research Institute of Infectious Diseases (FY13-20), Walter Reed Army Institute of Research (WRAIR #2098), and the Yerevan State Medical University covering Armenian institutions.

## Results

**Fig. 1:**
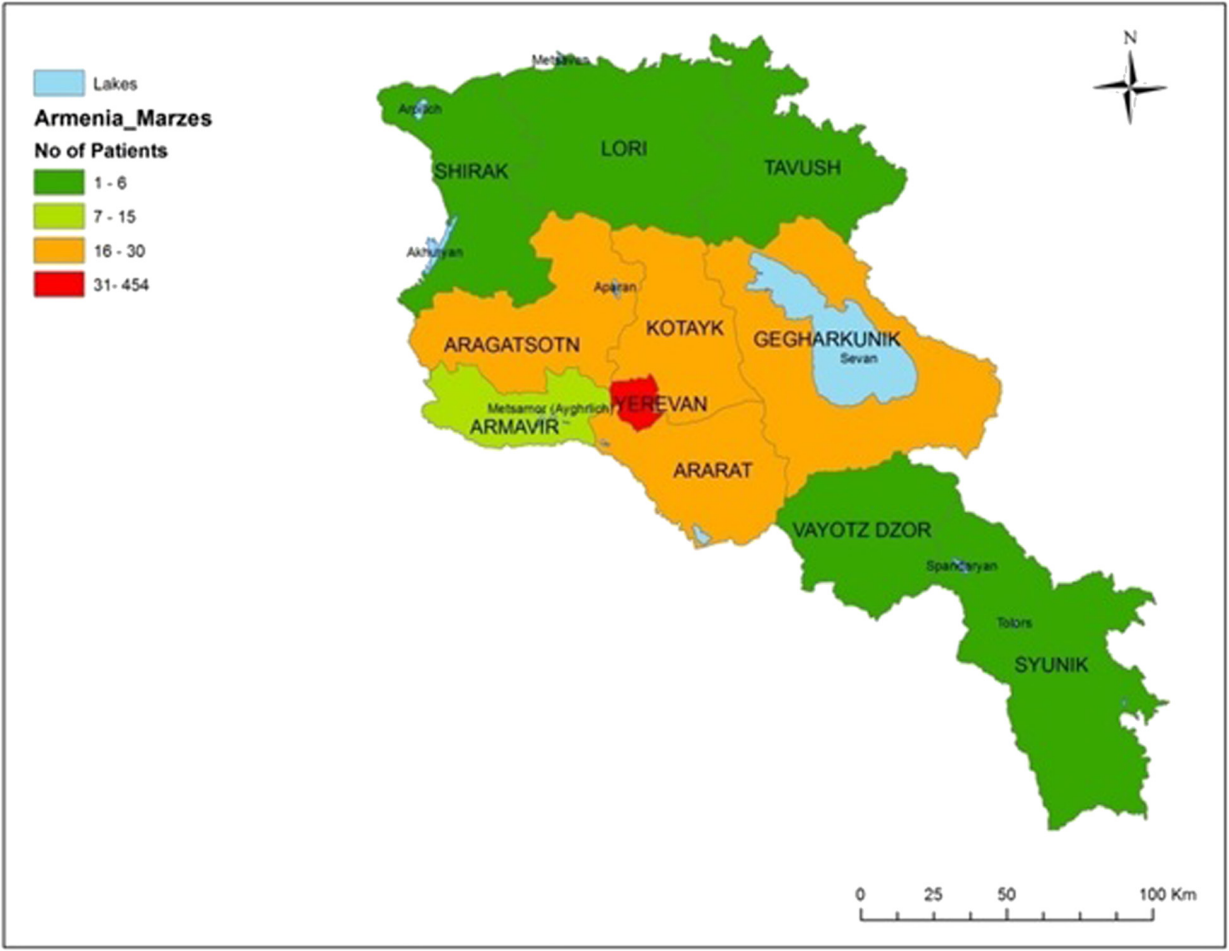
Geographic distribution of febrile hospitalized patients at the Nork Hospital by marzes, 2010–2012.

Of the 600 reviewed medical charts from hospitalized febrile patients at the Nork Hospital between 2010 and 2012, 51 % were males and the average age was 35.5 years (median = 30 years; Table 1). The majority of the patients were Armenian citizens (97 %), and 76 % were from Yerevan (Fig. 1). Patients from other regions (marzes) were admitted to the Nork Hospital, but to a lesser extent: Gegharkunik (5 %), Aragatsotn (4 %), Kotayk (3.9 %), Ararat (3.7 %), and Armavir (2.5 %). Most of the patient medical charts (78 %) did not register occupation; students comprised almost half (48 %) of those with known occupation. Of the reviewed charts, 71 % of the hospitalizations occurred between April and December.

**Table 1:** Demographic characteristics of febrile hospitalized patients at the Nork Hospital, 2010–2012

Feature	*n* (%)
Age group (in years)	
18–27	230 (44)
28–37	114 (22)
38–47	54 (10)
48–57	60 (11)
≥58	61 (12)
Gender	
Male	300 (51)
Female	284 (49)
Citizenship	
Armenian	580 (97)
Occupation	
Unknown	467 (78)
Student	64 (11)
Teacher	12 (2)
Manager	12 (2)
Healthcare personnel	8 (1)
Military	6 (1)
Other	30 (5)

Note: Denominators may vary due to missing data

Risk factor exposures for zoonotic and arboviral infections were not commonly recorded in the medical charts (Table 2). Based on available data, 5 % of the patients indicated that they had participated in agricultural activities, 4 % reported cattle exposure, and 1 % reported sheep exposure. Eight percent of the patients reported consumption of raw and unpasteurized milk products, and 11 % reported eating undercooked meat products. The percentage of patients who reported contact with a family member or with someone outside of the family with similar signs and symptoms before hospital admission was 11 and 15.2 %, respectively. Contact with water from rivers and ponds and visiting forests were rarely reported.

**Table 2:** Exposure history data of febrile hospitalized patients at the Nork Hospital, 2010–2012

Exposure history	*n* (%)
Contact with someone with similar signs	91 (15)
Contact with family member with similar signs	66 (11)
Consumption of undercooked meat products	64 (11)
Consumption of raw or unpasteurized milk	49 (8)
Exposure to animals	30 (5)
Traveled recently outside Armenia	30 (5)
Engagement in agricultural activity	29 (5)
Involvement in the slaughter of animals	17 (3)
Contact with animal abortus materials	12 (2)
Presence of rodents inside/around house	4 (0.7)
Contact with water from rivers and ponds	4 (0.7)
Visiting forests	2 (0.3)

Note: Denominators may vary due to missing data

Based on the inclusion criteria, all patients in our study population had fever. Fatigue (97 % of patients), diarrhea (55 %), nausea/vomiting (54 %), and shaking/rigors (52 %) were the most frequently reported clinical signs and symptoms. Fewer than 5 % of the patients reported skin lesions, pain behind the eyes, unusual bleeding, or neck stiffness. As to the physical examination findings, pallor (51 %), abdominal tenderness (43 %), pharyngeal injection (43 %), and lymphadenopathy (35 %) were the most common (Table 3). Other physical findings—including edema, jaundice, mental status change, joint effusions, conjunctival infection, skin lesions, neurological findings, bleeding, neck stiffness, and heart murmur—were reported in fewer than 5 % of the charts.

**Table 3:** Clinical signs and symptoms of febrile hospitalized patients at the Nork Hospital, 2010–2012

Reported symptoms	*n* (%)	Physical examination	*n* (%)
Fever	600 (100)	Pallor	386 (64)
Fatigue	579 (97)	Abdominal tenderness to palpation	309 (51)
Diarrhea	329 (55)	Pharyngeal injection	258 (43)
Nausea/vomiting	322 (54)	Lymphadenopathy	210 (35)
Shaking/rigors	314 (52)	Abdominal distention	147 (24)
Abdominal pain	278 (46)	Hepatomegaly	114 (19)
Headache	169 (28)	Splenomegaly	91 (15)
Excessive sweating	158 (26)	Respiratory crackles	86 (14)
Sore throat	131 (22)	Rash	79 (13)
Cough	81 (14)	Edema	30 (5)
Rash	88 (14)	Jaundice	27 (4)
Muscle soreness	76 (13)	Mental status changes	24 (4)
Joint pain	65 (11)	Joint effusions	23 (4)
Shortness of breath	40 (7)	Conjunctival injection	21 (3)
Skin lesion other than rash	15 (3)	Skin lesions	20 (3)
Pain behind eyes	9 (2)	Neurological findings	19 (3)
Unusual bleeding	6 (1)	Bleeding	18 (3)
Stiff neck	4 (1)	Neck stiffness	4 (1)
		Heart murmur	3 (1)

Note: Denominators may vary due to missing data

Almost one-quarter (24 %) of the patients were treated with antibiotics before admission to the Nork Hospital, being ceftriaxone (27.8 %), chloramphenicol (16 %), and trimethoprim–sulfamethoxazole (13.2 %) the antibiotics most commonly used. Antibiotic treatment was initiated at the Nork Hospital in 79.5 % (n = 477) of the patients, with ciprofloxacin (66 %) the most commonly used antibiotic. Of the patients treated with antibiotics at the hospital, 20 and 5 % of them received a combination of two and three antibiotics, respectively. Overall, the mean duration of hospitalization was 5.5 days. Few patients (*n* = 36; 6 %) had any complications during hospitalization.

With regard to final medical diagnoses (Table 4), intestinal infectious diseases (50.8 %) were the most commonly reported, followed by diseases of the respiratory system (11.2 %), infectious mononucleosis (9.5 %), chickenpox (8.3 %), brucellosis (8.3 %), and viral hepatitis (3.2 %). For purposes of reporting, the remaining diagnoses—malaria (*n* = 3), cutaneous anthrax (*n* = 2), leptospirosis (*n* = 2), fever of unknown origin (FUO, *n* = 2), rabies (*n* = 1), and rickettsiosis (*n* = 1)—were grouped into the category “Other”.

**Table 4:** Final diagnoses for febrile hospitalized patients at the Nork Hospital, 2010–2012

Final diagnosis	*n* (%)
Intestinal infectious diseases	305 (50.8)
Diseases of the respiratory system	67 (11.2)
Infectious mononucleosis	57 (9.5)
Chickenpox	50 (8.3)
Brucellosis	50 (8.3)
Viral hepatitis	19 (3.2)
Erysipelas	9 (1.5)
Other	43 (7.2)

### Brucellosis

In our study population, brucellosis was the fifth most frequently reported medical diagnosis. Of the 50 brucellosis cases, 37 (74 %) were in patients aged 18–47 years (Table 5). All of these patients first sought care at the Nork Hospital. We also found that brucellosis cases and non-brucellosis cases differed significantly by gender (*p* <0.0001) but not by age group (*p* = 0.449). The male-to-female ratio among brucellosis cases was 5 to 1; most cases were from the cities of Yerevan (28 %), Aragatsotn (20 %), Gegharkunik (16 %), and Kotayk (12 %). Of the patients with brucellosis, most (76 %) were diagnosed between the months of March and October.

**Table 5:** Demographics, exposure history, clinical manifestations, and risk factor analysis of hospitalized febrile patients with brucellosis, compared with non-brucellosis cases, at the Nork Hospital, 2010–2012

Feature	Brucellosis cases, *n* (%)	Non-brucellosis cases, *n* (%)	OR (95 % CI)	*p*-value
Demographic				
Age group (years)				0.449
18–27	19 (38)	245 (45)	–	
28–37	10 (20)	124 (23)	–	
38–47	9 (18)	53 (10)	–	
48–57	6 (12)	64 (12)	–	
≥58	6 (12)	63 (12)	–	
Gender				
Male	41 (84)	260 (47)	5.4 (2.5–11.8)	< 0.001
Female	8 (16)	276 (50)	Reference	
Exposure history				
Consumption of raw or unpasteurized milk	37 (76)	12 (3)	41.6 (806–201.5)	< 0.001
Exposure to animals	20 (39)	8 (1.5)	45.2 (18.4–110.9)	< 0.001
Engagement in agricultural activity	20 (41)	9 (1.6)	15.0 (4.0–55.7)	< 0.001
Involvement in the slaughter of animals	11 (22)	6 (1)	13.0 (2.8–64.2)	< 0.001
Contact with animal abortus materials	12 (24)	0 (0)	N/A	N/A
Clinical symptoms				
Excessive sweating	45 (90)	113 (21)	33.8 (13.1–87.1)	< 0.001
Joint pain	34 (68)	31 (6)	35.6 (17.7–71.4)	< 0.001
Muscle soreness	17 (34)	59 (11)	4.3 (2.2–8.1)	< 0.001

Note: Denominators may vary due to missing data. *OR* odds ratio, *CI* confidence interval. Out of those who reported having contact with animals, 15 (79 %) had contact with cattle and 4 (21 %) had contact with sheep. Sixty-one percent of the brucellosis patients did not report any contact with animals

At hospital admission, all patients with brucellosis had fever and fatigue. Other signs and symptoms also reported among brucellosis patients were excessive sweating (89 %), shaking (68 %), and joint pain (66 %). Brucellosis and non-brucellosis patients differed significantly on excessive sweating, muscle soreness, and joint pain (all *p* <0.0001). As to risk factors, consumption of raw or unpasteurized milk products, engagement in agricultural activities, exposure to animals, and participation in the slaughter of animals were significantly associated with brucellosis (Table 5).

## Discussion

In this chart review study, we found that most adult patients admitted to the Nork Hospital between January 2010 and December 2012 were Yerevan residents. We believe that the low percentage of patients from other regions (marzes) is probably because they sought medical care at their local healthcare facilities during the study period. In contrast, the high percentage of patients from Yerevan’s neighboring marzes (Kotayk, Gegharkunik, Ararat, and Aragatsotn) is due to the geographic proximity of these marzes to the capital city. The association between geographic proximity and healthcare seeking has been reported in other settings [14, 15].

Information on risk factor exposure for zoonotic and arboviral infections was very limited in the medical charts. Despite this limitation, one-quarter of the hospitalized febrile patients reported having had contact with someone—whether a family member or other individual—with similar signs or symptoms before admission. This finding suggests that direct, person-to-person contact or exposure to common infection sources may influence the transmission of diseases in Armenia. Infectious respiratory and intestinal diseases are among those that can be transmitted via person-to-person contact; for common zoonotic infections, such as brucellosis, the existence of another case of brucellosis in the home is an important risk factor for infection [16, 17]. Additional investigation is required in Armenia to determine the multiple sources for disease exposure and routes of transmission for emerging zoonotic infections.

In our hospitalized febrile population, the most commonly reported signs and symptoms were frequently observed among patients with acute enteric diseases [18]. We found that slightly more than half of hospitalized febrile patients were diagnosed with intestinal infectious diseases at the Nork Hospital during the study period. Our analysis did not reveal any association of specific signs and symptoms with other diagnoses also found in this chart review, such as respiratory tract infections, infectious mononucleosis, brucellosis, chickenpox, and viral hepatitis. Additionally, the frequencies of these diagnoses were similar across the study period (data not shown).

According to the medical charts, the most common zoonotic infection reported was brucellosis. Other infections, such as malaria, leptospirosis, cutaneous anthrax, rickettsial infection, rabies, and rat-bite fever were reported to a lesser extent. A diagnosis of FUO was also recorded in two hospitalized patients. Our findings indicate that these emerging and reemerging infectious diseases are circulating and are, to some extent, recognized by the healthcare system in Armenia. It is possible that the incidence and diversity of zoonotic and arthropod vector-borne infections are higher in rural regions of the country, where agricultural practices, as well as ecological and climatic conditions, offer an environment favorable for disease transmission. A recent study conducted in the Turkey–Armenia border area reported the presence of multiple mosquito species that are potential vectors of several pathogens [19]. Our findings may not represent the full diversity and burden of the zoonotic and arthropod vector-borne diseases in Armenia. Both limited laboratory capacity for these infections at the Nork Hospital as well as the geographic distribution of the cases—the majority of hospitalized patients in this chart review were Yerevan residents—might have had an influence on our results.

The male-to-female ratio, age, and gender distribution of brucellosis cases were similar to those reported in Georgia, a neighboring country [20]. Limited data indicate that the seasonal pattern of brucellosis found in our study, with hospital admissions peaking during March–August, was similar to the pattern reported in the 1960s in Armenia [21]. As to clinical findings, the most prevalent signs and symptoms (excessive sweating, joint pain, and muscle soreness) were also reported among brucellosis patients in Georgia, Iran, Yemen, and Tanzania [8, 20–23]. Additionally, the identified risk factors for brucellosis, such as engagement in agricultural activities, exposure to animals, and consumption of raw or unpasteurized milk, were consistent with those published elsewhere [8, 20–23].

Data from a large animal study indicated that high-risk areas for brucellosis transmission are the South and along the western and northwestern border of Armenia [24]. Most brucellosis patients in our study were from two areas not considered high risk: the cities of Yerevan and Aragatsotn, a province in western Armenia, 18 km from Yerevan. Therefore, it is likely that patients from high-risk brucellosis areas received medical attention in regional care centers. All brucellosis patients in our study were hospitalized for the first time; thus, data on relapses or chronic debilitating complications associated with brucellosis were not available from the medical charts. Additional brucellosis studies in high-risk areas are required for a comprehensive understanding of the epidemiology of this infection and its impact on population health in Armenia.

In Armenia, malaria was completely eradicated in 1963, but reemerged in the 1990s [25]. Data from the World Malaria Report indicate that the number of malaria cases significantly decreased between 1998 and 2005 [26]. After successful efforts to control and interrupt disease transmission—which included long-lasting insecticidal nets, insecticides, and better diagnostic techniques—the World Health Organization declared Armenia malaria free by the end of 2011 [27]. The three *Plasmodium falciparum* malaria cases (all of them males) found in our febrile population were imported cases from Africa. This fact shows that malaria-free countries should include, as a component in their strategic plans, coordinated efforts with endemic countries to control imported malaria as recommended by subject matter experts [28].

According to Nork Hospital statistics, 11 (37 %) of the confirmed cutaneous anthrax cases reported in Armenia during 2012–2013 received care at the hospital (unpublished data). Of these, six anthrax cases were hospitalized during our study period, but only two of them were eligible for study inclusion (the other four did not have a fever on admission). In the past, anthrax cases also occurred in the Shirak region (western Armenia) in 2001, and Kotayk and Gegharkunik in 2006. In March 2013, a confirmed case of skin anthrax was reported in a patient who had previously slaughtered cattle in a Georgian village [29].

Armenia had one case of rabies in the late 1990s [30]. According to national statistics data, one rabies case and eight leptospirosis cases were registered during 2009–2010 [31]. In this study, the patient diagnosed with rabies was a 36-year-old male who was admitted to the Nork Hospital in October 2011. The first human leptospirosis case documented in Armenia was registered in Aykadzor, a town in northwestern Armenia, in 1948 (unpublished data). Since then, sporadic outbreaks have occurred in Armenia, most of them in the western part of the country. The two leptospirosis patients found in this study were a 56-year-old male and a 49-year-old female, both admitted to the hospital in May–June 2010.

Since 1950, human cases of spotted fever group rickettsiosis (SFGR) have been reported in Armenia, and evidence from human and tick molecular studies suggests that SFGR is circulating in the country [5, 32]. The 58-year-old male patient who was diagnosed with rickettsiosis in this study was a resident of Armavir, a region in the western part of Armenia.

## Conclusions

Our results indicate that intestinal infectious diseases were the main causes of febrile illness, accounting for about one-half of hospital admissions; infections of the respiratory tract, infectious mononucleosis, and chickenpox together comprised approximately one-third of hospital admissions. Among zoonotic infections, brucellosis was the most frequently reported, followed by other emerging and reemerging diseases, such as rabies, rickettsiosis, leptospirosis, and anthrax. Although this study filled gaps in knowledge regarding the causes of febrile illness among hospitalized patients in the Nork Hospital, further integrated, prospective hospital- and population-based clinical and ecological research studies are required to provide more detailed information on the epidemiology of zoonotic and arthropod vector-borne diseases in Armenia.

## Abbreviations

FUO, fever of unknown origin; GIS, Geographic Information System; MoH, Ministry of Health; NCDCP, National Center of Disease Control and Prevention; SFGR, spotted fever group rickettsiosis; SPSS, Statistical Package for Social Sciences; WGS, World Geodetic System; YSMU, Yerevan State Medical University
